# Insights into the metabolism and behaviour of *Varroa destructor* mites from analysis of their waste excretions

**DOI:** 10.1017/S0031182018001762

**Published:** 2018-11-09

**Authors:** Francisco Posada-Florez, Daniel E. Sonenshine, Noble I. Egekwu, Clifford Rice, Robert Lupitskyy, Steven C. Cook

**Affiliations:** 1 Bee Research Laboratory, USDA-ARS Beltsville Agricultural Research Center, Beltsville, Maryland 20705, USA; 2 Department of Biological Sciences, Old Dominion University, Norfolk, Virginia 23529, USA; 3 Sustainable Agricultural Systems Laboratory, USDA-ARS Beltsville Agricultural Research Center, Beltsville, Maryland 20705, USA

**Keywords:** *Apis mellifera*, guanine, high-performance liquid chromatography, hypoxanthine, mass spectrometry, purines, xanthine

## Abstract

*Varroa destructor* mites (Acari: Varroidae) are harmful ectoparasites of *Apis mellifera* honey bees. Female foundresses of wax-capped pupal host cells and their daughters feed on host fluids from open wounds on the host's integument. Details of *V. destructor* mite nutrition are forthcoming, and little is known about the potential physical effects on hosts from mite feeding. Chemical analysis of waste excretions can infer details of animals’ nutrition. Here, chemical analysis by high-performance liquid chromatography/mass spectrometry (HPLC-MS/MS) of mite excretions showed that the purine content of *V. destructor* waste consists of guanine with traces of hypoxanthine. Traces of uric acid and caffeine were also detected. Concentrations of guanine attenuated over time and excretions collected from senescing mites did not contain detectable guanine. Non-reproducing individual female mites maintained *in vitro*, housed in gelatin capsules and provided a honey bee pupa, deposited an average of nearly 18 excretions daily, mostly on the host's integument rather than on the capsule wall. The weight and volume of excretions suggest mites can consume nearly a microlitre of host fluids each day. Compounded over 10 days, this together with open wounds, could lead to substantial water loss and stress to developing pupae.

## Introduction

*Varroa destructor* Anderson and Trueman (2000), hereafter *Varroa*, is an obligate ectoparasitic mite of the honey bee, *Apis mellifera,* and has been widely reported to be the major factor behind the continued poor health of this species, and collapse of honey bee colonies throughout most regions of the world (Allen-Wardell *et al*., 1998; Gallai *et al*., 2009; Nazzi and LeConte, 2016). Honey bees are essential for pollinating most domestic fruit and vegetable crops. Consequently, the continuing loss of honey bee colonies from *Varroa* parasitism (Varroosis) threatens a potential catastrophic loss of billions of dollars’ worth of crop production (Nazzi and LeConte, 2016; Rozenkranz *et al*., 2010). The threat of *Varroa* mites to the bee keeping industry has stimulated much research to understand *Varroa* mite biology and to develop methods for its control.

A search of the existing literature to date (2018) revealed relatively few articles relating to *Varroa* nutritional biology (see Evans and Cook, 2018 for a review). *Varroa* mites ingest a slurry of hemolymph containing dissolved nutrients, predigested host tissues and cellular components (Ramsey *et al*., 2018). Comparison of the fatty acid composition between *Varroa* and their honeybee hosts suggests that targeted feeding on host fatty acids may occur (Dmitryjuk *et al*., 2015). Other clues to the nature of *Varroa* nutrition include the presence of metabolic enzymes, including esterases, especially cholinesterases, carboxylesterases, alkaline and acid phosphatases, believed secreted by the mites' salivary glands (Dmitryjuk *et al*., 2014). Additional studies by Fraczek *et al*. (2009) revealed evidence of up to 19 hydrolases that were found in both honey bees and *Varroa* mites that feed on them, and the activities of certain hydrolases, e.g. glycosidases, were higher in the parasite than in the honey bee hemolymph. Also, the inhibition of the activity of bee hemolymph proteinases by *Varroa* extracts has been observed (Fraczek *et al*., 2012). A comprehensive study of the carbohydrate metabolism of *Varroa* detected two major glycogen metabolism enzymes, glycogen phosphorylase and glycogen synthase, along with other important findings regarding their lipid metabolism (Lopieńska–Biernat *et al*., 2013). Additional work has provided details of the sterol composition and synthesis capacity of host honey bees and *Varroa* mites (Hartfelder and Feldlaufer, 1997). Care should be exercised when interpreting *Varroa* nutrition based on the presence of certain metabolic enzymes, particularly those of host origin, as these may not be functional enzymes but simply represent undigested proteinaceous ‘food’ (see, Erban *et al*., 2015). In general, since the life history of *Varroa* is tightly linked to that of its host, understanding honey bee nutrition may provide basic insights into *Varroa* nutrition (see Wright *et al*., 2018 for a review). Finally, mite nutrition may also be supplemented by contributions from their microbiomes, which can differ in composition to that of their hosts (Hubert *et al*., 2016), or may affect the composition of host's microbiome (Hubert *et al*., 2017). Direct measures of microbial contributions to *Varroa* nutrition have not yet been undertaken.

Studies of ingesta is not the only means by which one can learn about animal nutritional biology; analyzing both the chemical composition of animal excreta and characterizing behaviours of excreta deposition, can inform researchers of an animal's nutritional balance (i.e. nutrient excesses), and provide insights into their metabolism, and possibly their hygienic behaviours. However, no studies have fully characterized the chemical makeup of *Varroa* excrement or investigated the excretory behaviours of *Varroa* during feeding, revealing that much still remains to be learned about *Varroa* nutritional biology and behaviour. Hence, here we characterize the chemical (purine) composition of *Varroa* excreta, observe the frequency of excreta deposition, and quantify and estimate mass and volumes, respectively, of individual deposits. Our findings will likely provide important insights into the metabolism of ingested bee tissues in the feeding mites.

## Materials and methods

### Collecting mites and honey bee pupae

Female *Varroa* mites and honey bee host pupae were obtained from honey bee colonies located on grounds of the United States Department of Agriculture-Agricultural Research Service (USDA-ARS), Beltsville Agricultural Research Center, Beltsville, Maryland, USA, that were untreated with acaricides to control *Varroa* infestations. Phoretic stage (*sensu lato*) mites used in experiments were collected from approximately 300 adult honey bees using the sugar roll method as described elsewhere (Dietemann *et al*., 2013). Mites washed of sugar were collected onto moist tissue paper lining a petri dish and transported to the laboratory. Honey bee host pupae were obtained from the same untreated honey bee hives as the mites. To do so, a frame having a large number of capped worker cells was removed, and cells gently opened to find white or pink-eye pupae, which were removed from their cells using soft forceps (Bioquip, USA) to minimize injury during extraction. All pupae were placed on moist tissue paper in a petri dish and transported to the laboratory. Back at the laboratory, both mites and pupae were inspected, and moribund mites and pupae showing evidence of injury were discarded. These same quality control procedures were implemented regularly for all mite and host collections. Using soft forceps, healthy pupae were transferred to gelatin capsules (size 0, Thermofisher Scientific, Waltham, Massachusetts 02451, USA). A single female mite was placed on pupa using a paint brush, then capsule closed by attaching the lid. Subsequently, the pupa and mite-filled capsules were placed in an Heratherm IGS-100 incubator (Thermo-Electric, Langenselbold, Germany) at 32 °C. and ~75% RH (Egekwu, *et al*., 2018).

### Excretory behaviours

A total of 12 *Varroa* were monitored over a 10-day experimental period. Each day the number of excretory deposits was recorded and whether each occurred on the capsule wall or on the host pupa. Additional measurements included weight and volume estimates of deposits made on, and collected from, the surface of hosts; weighed deposits were stored for subsequent analysis of their chemical composition. Excretory deposits on the capsule wall dissolved the gelatin, or were smeared, and thus not used for making physical measurements, nor included in the chemical analysis. To mitigate the risk of double counting deposits or compromise their chemical make-up, mites were transferred daily to a new capsule containing a fresh honey bee pupa. Statistical analyses of the differences in daily deposition rates and rates between capsule and pupa were determined by the General Linear Model and the Duncan Multiple Range test.

### Excreta deposit volume and weight

Each day, excretory deposits on the surfaces of the host pupae were observed under a 50X stereoscope and carefully removed using a fine heat-sterilized needle and then transferred to a stage micrometer (0.02 mm delineations) (MSWM2 stage micrometer, Microscope World Corp.). Deposits on host integument were often circular or subcircular in shape (Supplymentary Fig. S1); the diameter of each deposit was measured, and from this the volume of each deposit was estimated using the formula 4/3 *πr*^3^. Subsequently, excreta deposits were collected to a 1.5 mL microcentrifuge tube pre-weighed (± 0.1 mg) on an Ohaus Discovery Model DV-114C (Parsippany, New Jersey, USA) semi-microbalance. Excretion deposits collected from all pupae each day were amassed into the same tube and allowed to air dry for 3 h, after which the samples were re-weighed. The empty tube weight was subtracted from this final weight to determine the weight of the excreta deposited on host pupae. The same procedure was followed for each successive day of the experiment. Excreta deposits on the capsule wall could not be collected for weight/volume measurements (above). However, their total weight was estimated by taking the weight of a single deposit (calculated average individual excreta weight for deposits collected from pupae) and multiplying this value by the number of excreta deposited on the capsule wall.

### Chemical analysis of mite excreta

High-performance liquid chromatography coupled mass spectrometry with electrospray ionization (HPLC-MS/MS), together with multiple reaction monitoring (MRM) were used to detect and quantify purines in excreta samples. For this analysis, the more than 100 dried excretory deposits collected into separate microcentrifuge tubes on days 3, 6 and 9 from the host pupae were then dissolved in 1 mL 10% NH_4_OH added to the tubes. The three samples of *V. destructor* excreta extracts were submitted to the USDA-ARS Sustainable Agricultural Systems Laboratory, Beltsville, Maryland, USA for chemical analysis. In addition, authentic samples of guanine, hypoxanthine and xanthine (Sigma/Aldrich, St. Louis, Missouri, USA) were submitted for use as standards for comparison with the excreta samples. Prior to injection, the samples were diluted with methanol 1000-fold (samples 1 and 2) and 100-fold (sample 3). Subsequently, following the HPLC and mass spectroscopy procedures, the amount of guanine, hypoxanthine and xanthine in excreta samples was determined by comparison with purine standards.

Chromatographic separation of purines was performed on Waters Alliance e2695 HPLC (Waters Inc., Milford, Massachusetts, USA) using a reverse phase Phenomenex Luna C-8 column 4.6 × 150 mm, 3 *µ*m (Phenomenex, Torrance, California, USA). The mobile phase was a binary gradient with solvent A composed of 90% H_2_O and 10% 50 mm aqueous ammonium acetate and solvent B composed of 90% acetonitrile and 10% 50 mm aqueous ammonium acetate. The gradient was as follows: 0–4 min 100% A, 4–8 min linear change to 1.5% B, 8–11 min linear change to 20% B, 11–21 min 20% B, 21–23 min linear change to 100% B, 23–28 min 100% B, 28–30 min linear change to 100% A and 30–40 min 100% A. The total run time was 40 min. The flow rate was 0.3 mL min^−1^. The temperature of the column was 20 °C. The injection volume was 10 *µ*L.

The HPLC analysis was coupled with a Micromass model LC-Quattro Ultima triple quadrupole mass spectrometer with an ESI ionization (Waters Inc., Milford, Massachusetts, USA). The detection of purines was performed using MRM function in negative ionization mode for xanthine and positive ionization for hypoxanthine and guanine. The following parent–daughter ion transitions were used: M/z 151.1→108.1 (cone voltage 40 V, collision energy 15 V) for xanthine, M/z 137.1→119.1 (cone voltage 40 V, collision energy 19 V) for hypoxanthine and M/z 152.1→135.1 (cone voltage 40 V, collision energy 19 V) for guanine. Quantitation was performed by external standard methods without an internal standard. The source temperature was 140 °C and the desolvation temperature was 400 °C. Data were acquired and processed using MassLynx 4.1 software.

The concentration of guanine was determined using a 5-point calibration curve in the range from 0.05 to 1 ppm and linearity (*r*^2^ = 0.999).

## Results

### Excretory behaviours

**Fig. 1.**
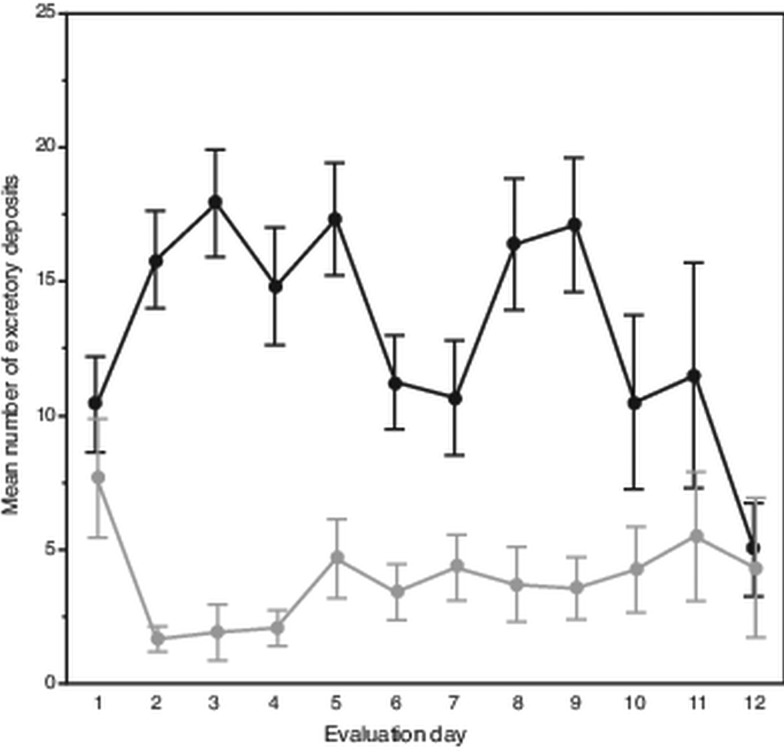
Daily number and location of female *Varroa* mite excretory deposits. Mean (± s.e.m) daily deposits of waste excretions made by mites observed over 12 days on integument of host honey bee pupae (upper dark line) and on inner wall of gelatin capsule (lower light line) housing mites and hosts.

Attrition of mites began on the eighth evaluation day until only six and three mites remained alive on days 11 and 12, respectively. Despite this, on average each day mites deposited 18 excretions. However, over the 12-day evaluation period, the mean number of deposits made each day varied, showing two separate peaks occurring on days 2–5 and 8–9 (Fig. 1). In total, a slight trend was observed showing fewer daily deposits over time (*F*_11,108_ = 1.69, *P* < 0.085). From the 120 total observations made of excretory behaviour, mites deposited excretions predominately on the body of their hosts (*χ*^2^ = 118.70; df = 1, *P* < 0.0001); on average each day, mites deposited about 14 excretions on the pupae *vs* only about 4 on the gelatin capsules.

### Excreta total weights and volumes

**Fig. 2.**
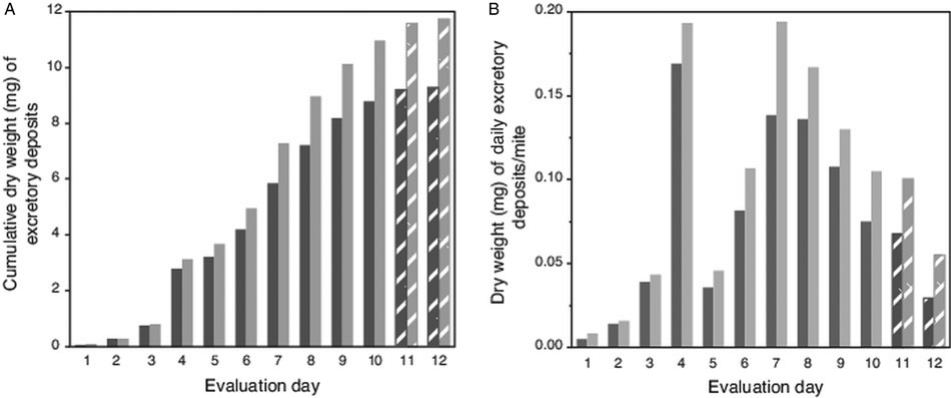
Weight of cumulative total, and estimated weights of individual excretory deposits from female *Varroa* mites. (A) Cumulative weight of excretory deposits collected over 12 days from integument of host pupae (dark bars) and total weight of deposits (light bars) including estimated weights of deposits on capsule walls. (B). Estimated total weight of individual excretory deposits made daily by individual mites. Textured bars in both (A) and (B) represent estimated weights (see text).

Because high attrition of mites in last 2 evaluation days led to numbers of excretions on pupae falling below a preset threshold (>100 total deposits from all mites each day; Table 1), no deposits were accumulated from pupae for weighing on evaluation days 11 and 12. However, for these days, the number of deposits were multiplied by the average weight of a single deposit from host pupae (5.9 *µ*g ± 1.4 *µ*g s.e.m). Estimated weights of deposits on capsule wall (using a number of deposits and estimated weight of a single deposit) was 79% (±3% s.e.m) accurate when extrapolating from actual weighed excreta removed from host's body. For this complete dataset, the trend for the cumulative excreta weights shows a positive continuous accumulation over the 12-day period without any lapses (i.e. deposits were not absent on any evaluation day) (Fig. 2A). The estimated weight of excreta deposited daily per mite is shown in Fig. 2B; excreta weights peaked on days 4 and 7 (each nearly 0.2 mg), then declined steadily to day 12.

**Table 1.** Number of excretory deposits made by individual female *Varroa* mites on either their honey bee pupa host or wall of gel capsule housing both pupa and mites over a 12-day monitoring period

Evaluation day	Number of mites	Number excretory deposits	
		Pupae	Capsule
1	12	125	92
2	12	190	20
3	12	215	23
4	12	178	25
5	12	208	56
6	12	135	41
7	12	128	52
8	10	164	37
9	9	154	32
10	8	84	34
11	6	69	33
12	3	15	13

**Fig. 3.**
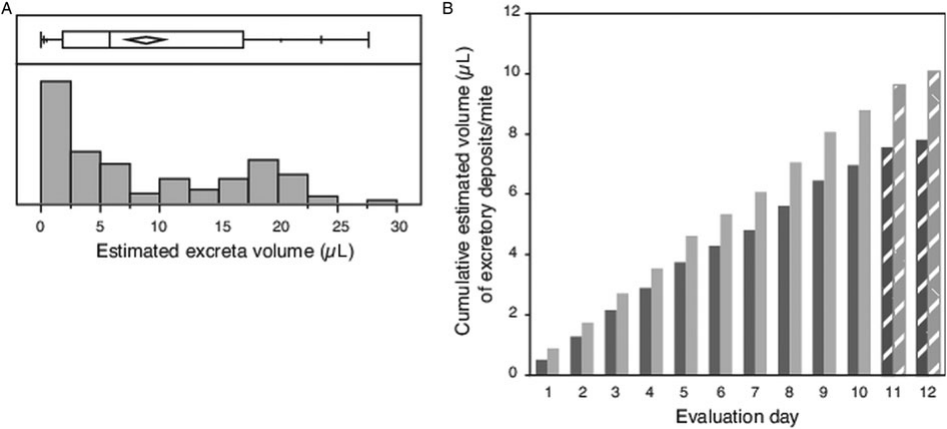
Volume of individual excretory deposits from female *Varroa* mites. (A) Box plot giving range, quartiles and median of excretory deposit volumes calculated from the measured diameter of *N* = 102 circular/semi-circular excretory deposits, and the frequency distribution of these calculated volumes. (B) Cumulative volume of excretory deposits from individual mites over 12 days, estimated from the mean calculated volume of individual deposits and number of deposits made daily per mite. Textured bars in (B) are estimated data extrapolated from measured excreta from days 1 to 10.

The diameter was measured for an average of 17 individual circular/subcircular shaped excretory deposits (haphazardly selected subset of total deposits collected each day 1–10), resulting in calculated volumes ranging from a minimum of 0.002 *µ*L to a maximum of 0.153 *µ*L, with many deposits having small volumes (Fig. 3A). The mean volume of each deposit measured (*N* = 102) was 0.049 *µ*L ± 0.004 s.e.m. If mites excreted 18 such deposits each day, compounded over the 12-day evaluation period, each mite would have consumed an estimated 10 *µ*L of host fluid (Fig. 3B).

### Chemical analysis of mite excreta

**Fig. 4.**
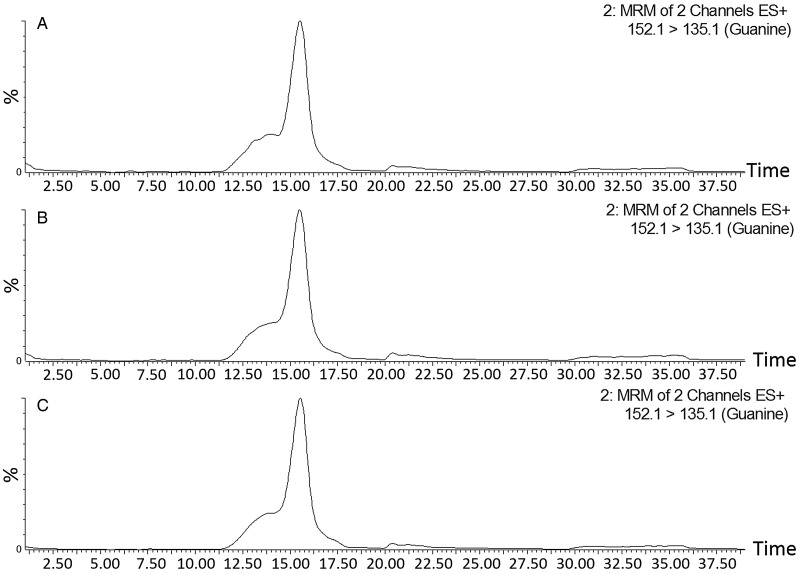
LC-MS analysis of purine standards and *Varroa* mite excreta samples amassed at day 3 (sample 1), day 6 (sample 2) and day 9 (sample 3), of a 12-day monitoring period. (A) LC-MS/MS chromatogram for MRM transition M/z 152.1→135.1 of sample 1 at 2.54 ppm. (B) sample 2 at 1.59 ppm, and (C) guanine standard at 0.25 ppm. Guanine was not detected in sample 3.

Chemical analysis of the excretory deposits revealed the presence of guanine and traces of hypoxanthine, but no evidence of xanthine. The mass spectrum obtained from sample two closely matched the mass spectrum for the guanine standard (Fig. 4 and Supplymentary Fig. S2). For the other samples, slight deviations from the standard in the spectra are likely due to impurities in the samples. In addition, analysis of full scans and comparison with published mass spectra revealed the presence of uric acid and caffeine in all three samples (data not shown), and possibly an undetermined purine (mainly in samples 2 and 3). Guanine content in samples 1 and 2 was 0.23 and 0.18 mg mL^−1^, respectively. No evidence of guanine was detected in sample three. Analysis of the HPLC chromatogram and mass spectrum for MRM transition also showed guanine in both the standard and samples one and two. The MRM results are considered definitive; these results identified both the unique collision-induced fragment of guanine (the 135 m z^−1^ ion) determined from the standard, and the same 152 > 135 m z^−1^ ion chromatogram retention time as the standard. This retention time for guanine was also unique compared with the ionization fragments and retention time for hypoxanthine. Trace hypoxanthine identified in samples was not quantified.

## Discussion

To gain a better understanding of *Varroa* nutrition and excretory behaviour, we both characterized the purine composition of their excreta and described their excretory behaviours while housed with honey bee pupa hosts in the laboratory. One method to understand the nutritional biology and physiology of organisms is to identify the chemical components and their relative abundance in waste excretions. Guanine, hypoxanthine, xanthine and other purines serve as the nitrogenous wastes in ticks and other acarines (Kniest, 1995; Sonenshine, 2014); high levels of purines in excreta may indicate a protein-rich diet and/or a high level of protein/nucleic acid metabolism. Guanine was also found to be abundant in the excretory waste of house dust mites, *Dermatophagoides pteronyssinus* and *D. farinae* (Quiox *et al*., 1993). In contrast, for blood-feeding insects, such as kissing bugs (Triatominae), the primary nitrogenous waste is in the form of uric acid salts, e.g. potassium urate (Graça-Souza *et al*., 1999; Bradley, 2003). Previously, the waste excretions of *Varroa* mites were reported to consist of 95% guanine (Erickson *et al*., 1994). Our analyses indicate that the guanine content of excreta attenuated over time; the percentage of guanine in excreta collected on days 3 and 6 was 48.9 and 18.4%, respectively, and no measurable guanine was measured in excreta collected from day 9. In relation to this, we noted that mites began dying in large numbers after the 10-day monitoring period (coinciding with depletion of white material visible in the digestive tract of mites; Supplymentary Fig. S3). Although the reason for sudden increase in mite mortality is unknown, the attenuation of guanine content in excreta suggests that at latter days *Varroa* decreased feeding on its host, perhaps due to physical and/or physiological costs associated with making a new feeding hole each day. However, the estimated weight/volume of individual excreta did not show a corresponding decrease, suggesting that compounds other than those measured increased in prevalence. Indeed, unidentified purines appeared more abundant in the later samples (as noted above), raising the possibility of failure of metabolic processing as a major reason for mite mortality.

The chemical composition of *Varroa* excretions, besides providing basic information on mite nutritional biology and physiology, also can provide information for applied research. For example, in other acarines guanine can act as an arrestant pheromone alerting conspecifics to favourable locations to find new hosts (Leahy *et al*., 1975; Gray, 1987; Dusbabek *et al*., 1991; Allan and Sonenshine, 2002; Sonenshine *et al*., 2003). Guanine may also serve as an assembly pheromone for *Varroa*, guiding other mites to infested brood (Sammataro *et al*., 2000; Yoder and Sammataro, 2003), or in an applied scenario to traps and/or poison-laced baits. In a different study, other authors have suggested that the presence of guanine in honey bee hives might act as a diagnostic tool for identifying infestations of the honey bee tracheal mite, *Acarapis woodi* (Mozes-Koch and Gerson, 1997).

In terms of excretory behaviours, our data show that female mites defecated nearly four times more frequently on their hosts than on the capsule wall. This finding contrasts with the behaviour reported by others, who found that the mites preferred to deposit wastes in the posterior part of the brood cell (Nazzi and LeConte, 2016) or on the sides of the cells (Calderón *et al*., 2009, 2012; Dieteman *et al*., 2013). This behaviour may be adaptive, possibly to promote hygiene within the confines of the sealed host cell. Also, Calderón, *et al*. (2012) hypothesized that reproductive females do not deposit wastes on the surface of hosts, and those that did so had reduced fecundity. Santilián-Galicia *et al*. (2008) reported that the honey bee pathogen Deformed wing virus was found located in large, dense spheres presumed to be excretory waste within the midgut of *Varroa.* If true, this may provide a better understanding of how host health is compromised, thus reducing mite fecundity. We also found evidence of granular material, presumably guanine, in the midgut of female mites (Supplymentary Fig. S3), similar to that shown by Erban *et al*. (2016) in the stored product mites, *Tyrophagus putrescentiae*.

Monitored mites each made on average nearly 18 excretory deposits daily. The number of excretory deposits was similar each day over the monitoring period, suggesting female mites maintain a consistent feeding regiment. The mean volume per deposit, 0.049 nL ± 0.044, times an average of 18 excreta deposits for each mite per day, represents an average of 10 *µ*L of waste excreted over 12 days. Comparing excreta volume to mass, which was estimated as >1 mg dry weight over 12 the water-saving properties of purines becomes apparent. Nevertheless, water loss from mite feeding, together with wounds remaining open *via* mite released anticoagulating proteins (Yang and Cox-Foster, 2005) can equate to a substantial moisture loss from hosts, which may be injured as a result. Frequent daily excretion observed here supports data showing mites feed frequently from their hosts (Calderón *et al*., 2009), or have a high metabolic turnover. Moreover, our data suggest that mites do not store any ingested food, but ingest relatively large volumes of food daily and frequently excrete the waste products. These behaviours and any associated internal anatomy might be necessary given the tight and crowded quarters inside capped honey bee pupa cells (Calderón *et al*., 2009).

The high nitrogen content of *Varroa* excretions, together with the frequent deposition of large volumes of excreta, is reminiscent of filter-feeding insects, such as aphids, which strain carbohydrate (sugar) rich plant phloem for the trace amino acids and other nutrients it contains and expel sugar-rich honeydew. As presumably non-reproducing (or growing) phoretic-stage mites were used in our experiments (no eggs were noted at any point during monitoring period), it is unclear what nutrients, if any, mites may be straining from ingested host fluids. If female mites were actively reproducing, it could be predicted that the frequency, and perhaps volume and chemical composition of excretory deposits may differ; ingested protein may not be readily discarded, but rather shunted into forming several large, nutrient-dense eggs. Future work should determine the complete chemical composition of *Varroa* excreta, to provide clues of not only protein metabolism, but that of other nutritive substrates, such as lipids. These data, together with information of differences in feeding, and thus excretory behaviours between ‘phoretic’ and reproducing ‘foundress’ female mites, may help to distinguish the nutritional requirements of mites in these contrasting life stages. Finally, future work should also investigate the unknown, specific contributions of the mite's microbiome and host enzymes, if any, to the nutrition of *Varroa* mites.

## Supplementary material

For supplementary material accompanying this paper visit https://doi.org/10.1017/S0031182018001762. click here to view supplementary material
