# Supplementary figures and images for: Insights into the metabolism and behaviour of Varroa destructor mites from analysis of their waste excretions
Source: Parasitology. 2018 Nov 9;146(4):527–32. doi: 10.1017/S0031182018001762 (PMC6425362; doi:10.1017/S0031182018001762)

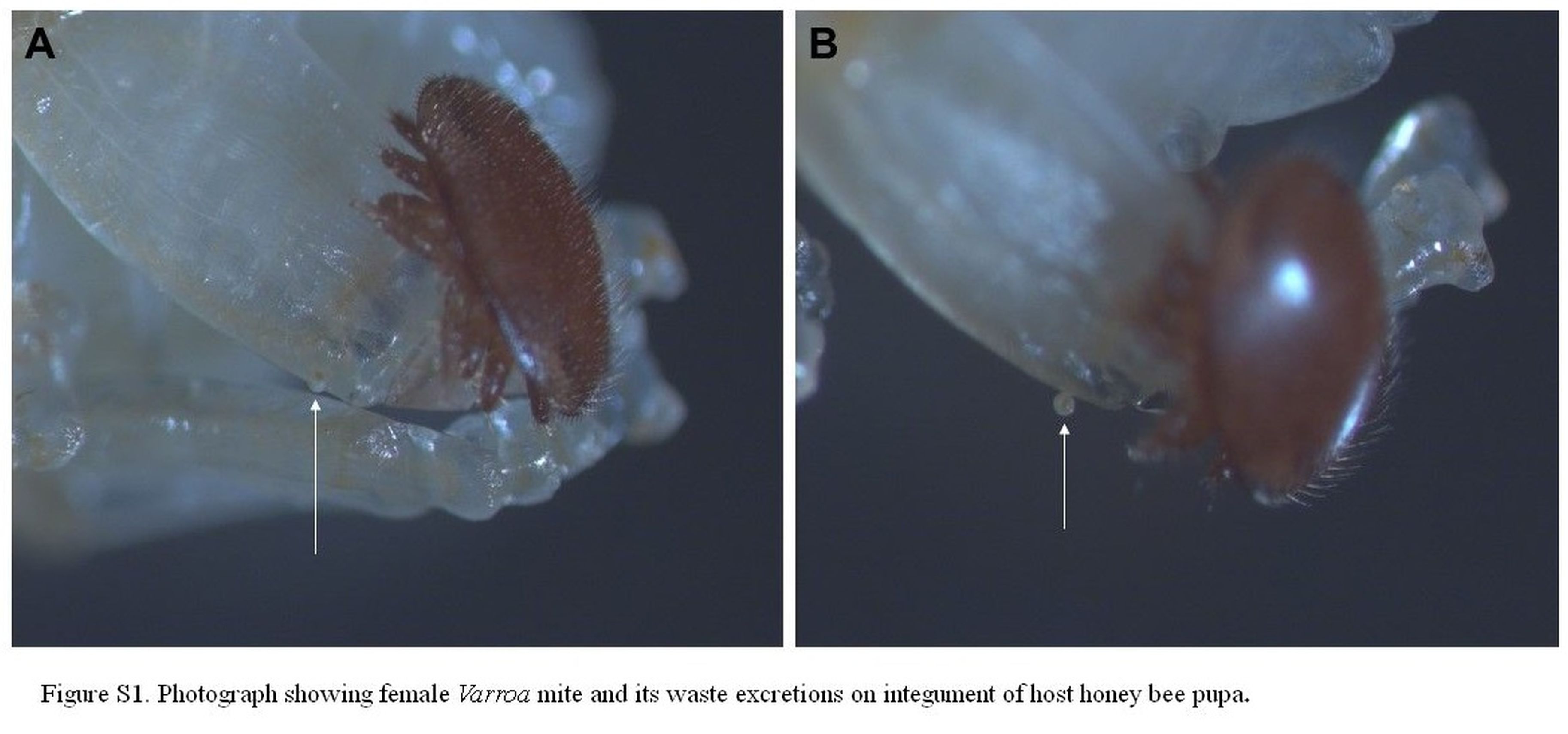

Supplement: Supplementary file 1 [file S0031182018001762sup.zip › S0031182018001762sup001.jpg]

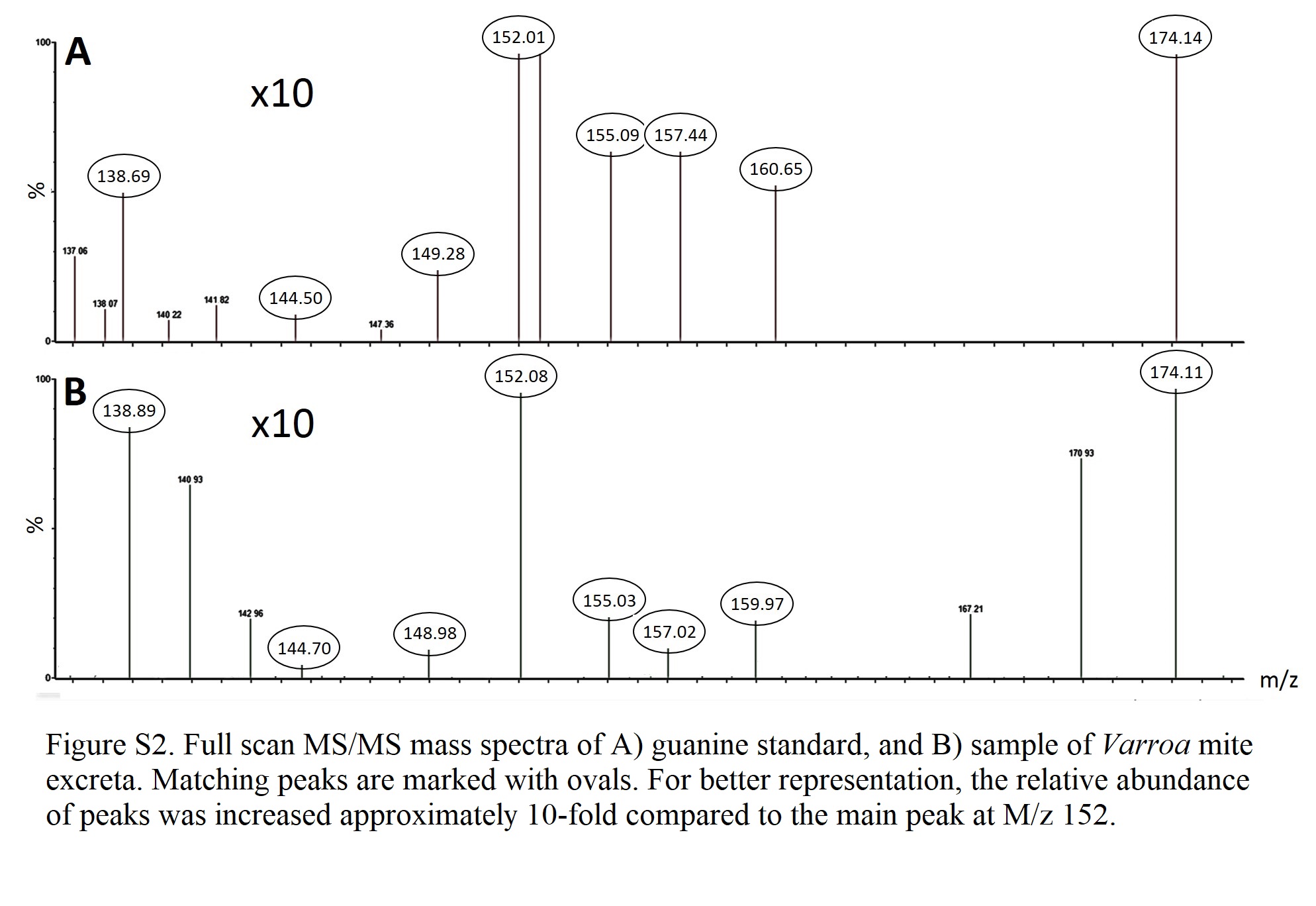

Supplement: Supplementary file 1 [file S0031182018001762sup.zip › S0031182018001762sup002.jpg]

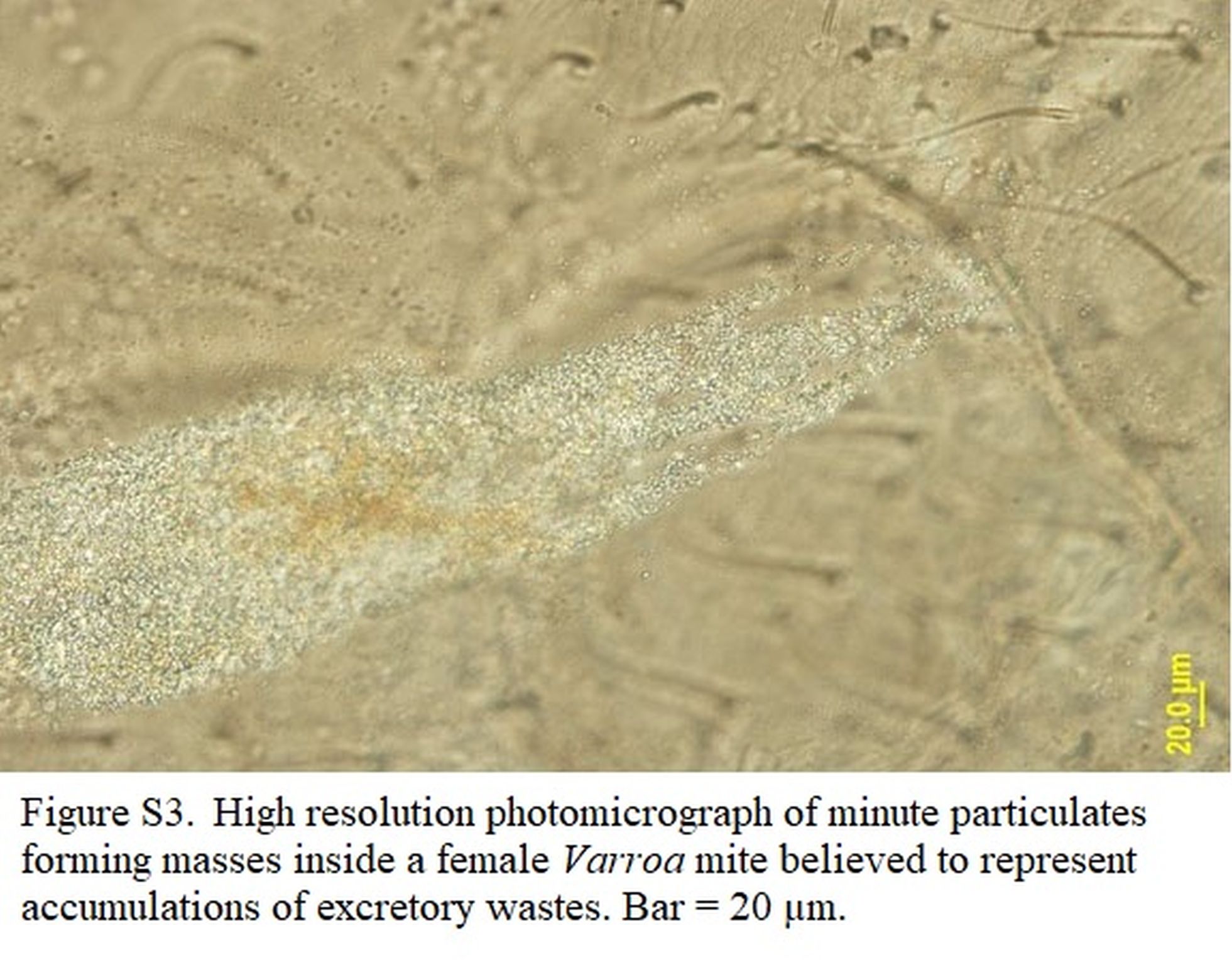

Supplement: Supplementary file 1 [file S0031182018001762sup.zip › S0031182018001762sup003.jpg]
